# “You Can Get Away with Anything Here… No Justice at All”— Sexual Violence Against U.S. Indigenous Females and Its Consequences

**DOI:** 10.1007/s12147-021-09291-6

**Published:** 2021-10-03

**Authors:** Catherine E. McKinley, Hannah Knipp

**Affiliations:** 1 Tulane University School of Social Work, New Orleans, LA, USA

**Keywords:** American Indian, Native American, Indigenous, Sexual violence

## Abstract

Sexual violence against Indigenous women has long been used as a tool of colonial violence and conquest. As a contemporary form of historical oppression that may drive associated health and mental health inequities, Indigenous women in the United States experience sexual violence at greater levels than the general population and at and twice the rate of Indigenous men. We use the Indigenous framework of historical oppression, resilience, and transcendence (FHORT) to understand Indigenous women’s experiences of sexual violence and how it differentiates across ecological outcomes related to health and wellness. This exploratory sequential multimethod study with 563 participants (*n* = 436 qualitative and *n* = 127 quantitative survey participants) qualitatively explores how Indigenous peoples describe sexual violence and quantitatively investigates key differences across ecological outcomes of wellness related to sexual violence, including alcohol use and post-traumatic stress disorder (PTSD). Results indicated that all participants (100 percent) who reported sexual violence were women. Thematic analysis of qualitative results revealed the themes related to familial, non-familial, and the historical oppression of a lack of accountability for perpetrators. Quantitative *t*-Tests results revealed that experiencing sexual violence was associated with significant differences across ecological dimensions of wellness including (a) structural: higher historical oppression, historical loss, oppression, and discrimination; (b) relational: higher adverse childhood experiences and stressful life events and lower family resilience and social support; (c) spiritual: lower spiritual-well-being and life satisfaction; and (d) psychological/behavioral: higher levels of alcohol use, PTSD, and lower levels of psychological resilience. Thus, sexual violence profoundly affected Indigenous women.

Sexual violence against Indigenous women has long been used as a tool of colonial violence and conquest. Given that women were revered, held sacred, and held great authority in many female-centered and matriarchal Indigenous societies, it is not surprising that women were targeted as leaders during colonization [12, 13, 16]. Targeting these authority figures was essential to controlling and conquering Indigenous peoples as a whole. Although sexual violence disproportionately affects all women [64], unlike non-Indigenous women, sexual violence against Indigenous women occurs within the context of the postcolonial historical oppression. As a contemporary form of historical oppression that is perpetuated through impunity to perpetrators, Indigenous women in the United States experience disproportionate rates of sexual violence. This violence my drive many associated health inequities. We examined Indigenous women’s experiences of sexual violence and how this violence may differentiate key aspects of wellness across structural, relational, psychological, and spiritual dimensions.

Indigenous people of the U.S. (we limit the scope of this inquiry here to American Indian/Alaskan Natives, but these populations can also include Native Hawaiians and Indigenous peoples from U.S. and associated Pacific Island flagship territories) are disproportionately impacted by sexual violence [2, 58, 59]. The higher rates of sexual violence and lack of accountability for perpetrators is a contemporary form of historical oppression [16], which may drive associated health inequities, including post-traumatic stress disorder (PTSD) and problem alcohol use. Indigenous women report approximately twice the prevalence of lifetime sexual violence (56 percent) compared to Indigenous men (28 percent), as well as higher rates than White women (49 percent) and White men (21 percent) [58]. Given their elevated rates, the scope of this inquiry is limited to Indigenous women, who are more likely than Indigenous men to be hit, injured, and need medical attention [2].

Experiences of sexual violence contribute to many of the health inequities experience by Indigenous women, such as poor mental health (PTSD, acute stress disorders, depression, sleep disorders, anxiety, suicidal ideation) decreased sexual urges/pleasure, increased sexual risk behaviors, problem alcohol and drug use, and physical health problems [18, 61]. To ameliorate sexual violence and associated health problems, Indigenous women’s experiences of sexual violence must first be understood. More understanding as to whether such experiences may differentiate across key aspects of ecological dimensions of wellness is also salient, including known health disparities related to alcohol use and PTSD.

The Department of Justice found that rates of sexual violence are approximately 2.5 times higher for Indigenous women than White, African American, or Asian American women [2]. Children and youth are also impacted. Indigenous youth are twice as likely to be forced into sex than White youth, as found in one study of urban youth [59] and 17 percent of Indigenous women reported their first sexual experience was nonconsensual [60]. One study found that 22 percent of Indigenous women and 15 percent of Indigenous men experienced childhood sexual abuse [26].

Due to a reversal of the prior matriarchal and female centered gender dynamics of many Indigenous communities and the impunity for perpetrators [12, 13, 16], sexual violence represents a continuation of the historical oppression introduced in colonization and perpetuated through continued act of such violence and trauma. Unlike the general population, Indigenous women belong to 574 federally recognized tribes in the United States, more than 60 state-recognized tribes [54] and those tribes still seeking recognition. Moreover, the United States entered into treaty agreements in exchange for its land, and in those treaty agreements entered into an assurance to provide for tribal members’ health and well-being [10]. Thus, sexual violence and its consequences directly undermines the assurance of Indigenous women’s health and well-being.

Further, historic and contemporary federal laws create a multitude of barriers for achieving justice for survivors of sexual assault [1, 23]. The exorbitant rate of sexual violence indicates the United States government not only continues to fall short of this trust agreement, but also impedes the path to justice. Although we know much about the experiences and impact of sexual violence within the general population, less is understood about Indigenous women’s experiences and associated consequences. We approach this work with the framework of historical oppression, resilience, and transcendence (FHORT), which was developed with Indigenous communities to understand risk factors for sexual, physical, and other forms of violence [16].

## The FHORT and Sexual Violence against Indigenous Women

The FHORT is a liberatory, culturally responsive, and anti-oppressive, inclusive theoretical framework that helps to contextualize Indigenous women’s experiences of sexual violence in a context of patriarchal and colonial historical oppression [16]. The FHORT’s ecological framework characterizes wellness (attaining balance across structural, relational, psychological/behavioral, and spiritual dimensions) by examining intersecting and multi-level risk and protective factors related to sexual violence [16]. The FHORT was developed to understand and explain violence against Indigenous women and children, and in particular, intimate partner violence, which includes sexual violence [16]. It is extended in this inquiry to focus solely on sexual violence.

The FHORT aligns itself to diversity, equity, and inclusion frameworks while simultaneously employing a trauma-informed lens. In contrast to cultural competency models, which are critiqued for their limited focus on the acceptance of various cultures, the FHORT is better aligned to a cultural humility model [32]. Cultural humility requires researchers to both deeply understand structural oppression and maintain an unwavering commitment to social justice [32]. Further, practitioners and researchers must adopt a trauma-informed perspective to work with Indigenous communities effectively and ethically [70]. The FHORT practices cultural humility [32] and accomplishes the primary goal of trauma-informed research–recognizing the trauma [29] by highlighting historical oppression as a focal point. Further, by pairing this understanding with a commitment to resilience and transcendence, the FHORT demonstrates cultural humility by prioritizing social justice [32].

Indigenous women are distinctive from non-Indigenous groups as experiences of historical oppression encompass patriarchal colonialism [36] including the intersections of colonialism, racism, and sexism. Hilary Weaver (2009) posits that higher rates of violence in Indigenous communities are the result of higher patriarchal colonialism norms, which over time, have been internalized by Indigenous communities [69]. These internalized norms can perpetuate violence from external forces, such as a lack of justice for perpetrators, as well as internal forces, such as internalized patriarchal norms passed down intergenerationally through horizontal violence and sub-oppression of fellow tribal members [16]. According to the FHORT, through the mechanisms of internalized oppression, one may adopt the devaluing views against women, which may give rise to higher rates of sexual violence toward women [16]. This higher rate of violence is exacerbated by impunity for perpetrators and a lack of justice [14]. Historical oppression of this sort undermines the resilience of matrilineal Indigenous societies.

Indigenous women’s experiences of sexual violence are a historic and contemporary form of patriarchal *historical oppression*—“the chronic, pervasive, and intergenerational experiences of oppression that, over time, may be normalized, imposed, and internalized into the daily lives of many Indigenous people” (p. 38) [16]. Indeed, from the perspective of the FHORT, the colonial and dehumanizing tools of invasion, sexual conquest, manipulation, and division parallel those tactics of power and control found in violence against women [12, 13, 16, 34]. Before colonization, Indigenous gender relations in the U.S. Indigenous communities were characterized by humanizing, egalitarian, complimentary, and cooperative gender norms where women held high status and respect [12, 13, 16].

According to the FHORT, historical oppression is societal level risk factor, extending the concept of historical trauma to include current and past forms of oppression. Colonial historical oppression disrupted the once matrilineal and woman-centered gender roles to be replaced with Western forms of the patriarchy (men subjugate women) and Eurocentrism (White’s to subjugate non-Whites) undermining gender relations and the status of Indigenous women [9–11]. This reversal in gender relations imposed patriarchal gender roles that placed women at risk for sexual violence. Gender inequities and patriarchal gender roles contribute to violence against women [39], along with impaired relationship quality and increased conflict [11, 44, 55]. Moreover, Indigenous men may have constricted social mobility due to intersecting oppressions [40]. As such, they may experience more gender role strain from being unable to fulfill prescribed patriarchal roles, which may lead to lashing out and horizontal violence including establishing control in more extreme ways, such as sexual violence [16]. Recent work confirmed historical losses in the forms of loss of values that protected and provided respect for women as well as a loss of holding perpetrators accountable. This loss of values led to the reversal of matrilineal gender roles, which were replaced with dehumanizing patriarchal roles that subjugated women. The loss of accountability indicated community members to keep things silent or not intervene in cases of violence, inadvertently protecting the perpetrator and precluding accountability [13].

**Fig. 1:**
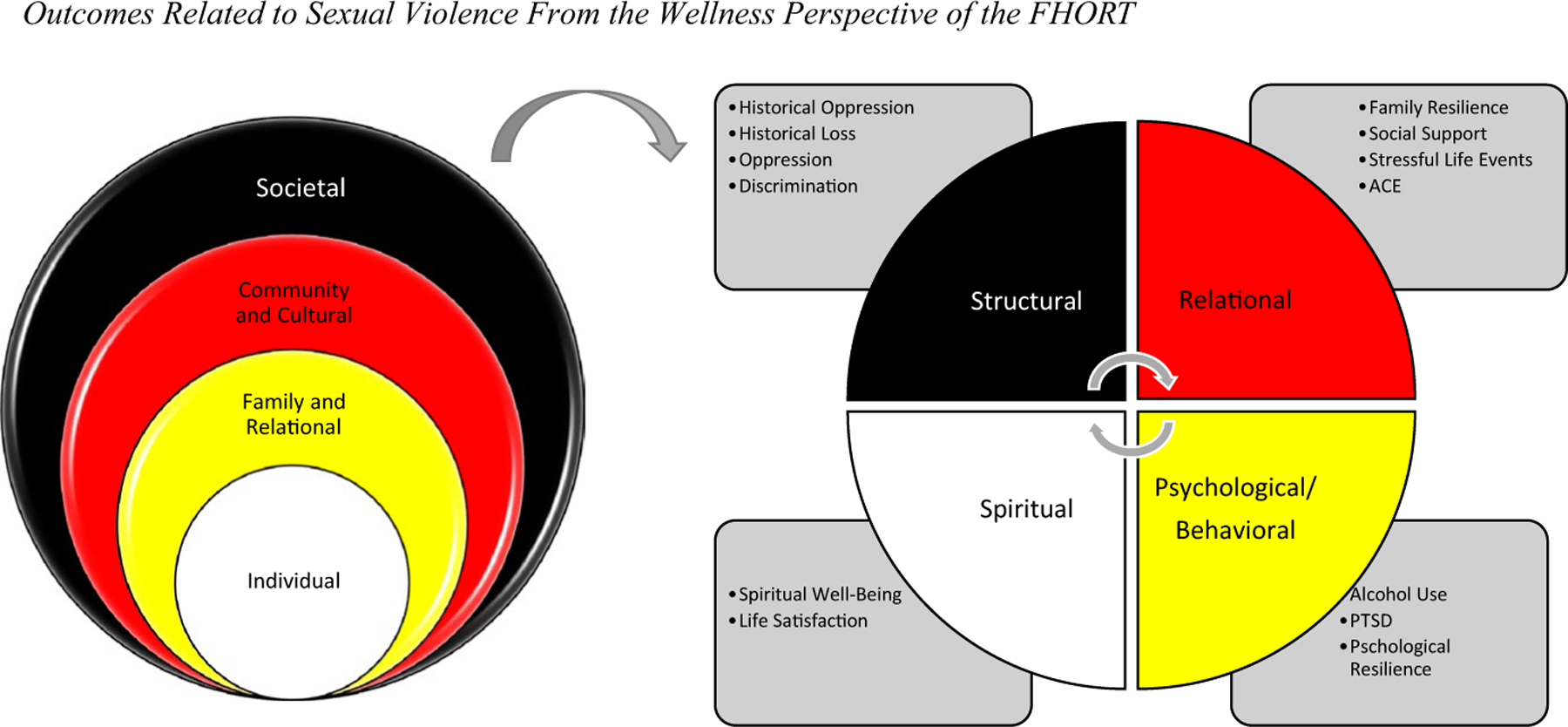
Outcomes Related to Sexual Violence From the Wellness Perspective of the FHORT. *Note* According to the FHORT, ecological risk and protective factors occur across societal, cultural and community, family and relational, and individual levels. Sexual violence against Indigenous women and girls is considered an outcome of centuries of historical oppression. This oppression began with colonization, has been internalized into the oppressive patriarchal norms targeting women, and results in the disproportionate rates of sexual violence against Indigenous women today. We investigate structural (historical oppression, loss, and discrimination) relational (family resilience, social and community support, stressful events, and adverse childhood experiences (ACE).) psychological/behavioral (alcohol use, PTSD, and psychological resilience) as well as spiritual (spiritual well-being and life satisfaction) dimensions of wellness. In this way, we investigate key concepts of the FHORT (namely historical oppression, resilience (family and psychological) and transcendence (spiritual dimensions of wellness)

Historical oppression has set the stage for the adverse social and health outcomes, such as sexual violence; yet Indigenous people have striven and demonstrated resilience (recovering from adversity) and in some cases, even transcendence (experiencing greater well-being than if adversity had not been imposed) [16]. Figure 1 portrays the FHORT as it relates to sexual violence against Indigenous women.

In this inquiry, we first, qualitatively explore Indigenous women’s experiences of sexual violence. Next, we quantitatively examine whether experiences of sexual differentiate key aspects of wellness, which are also primary tenets of this framework—namely historical oppression (and loss, general oppression, and discrimination) resilience (family, psychological, as well as social support) and transcendence (spiritual well-being and life satisfaction). We strive to understand both the lived experiences of sexual violence in participants’ own words, and how sexual violence may differentially be associated with key outcomes that may drive many healthy inequities experienced by Indigenous women.

## Impact of Sexual Violence

The impacts of childhood sexual abuse, sexual assault, and rape have been studied extensively [18, 46, 61]. Following an assault, common outcomes include poor mental health (PTSD, acute stress disorders, depression, sleep disorders, anxiety, suicidal ideation) decreased sexual urges/pleasure, increased sexual risk behaviors, substance use and physical health problems [18, 61]. For survivors of childhood sexual abuse, lack of support and negative responses from parents resulted in worse outcomes, long-term [46]. Additionally, women who were sexually assaulted in childhood are twice as likely to be assaulted as adults [61].

Less is known about Indigenous women specifically, although some quantitative studies have focused specifically on the impacts of sexual violence on this population [4, 7, 9, 26, 27]. Long-term impacts of sexual assault for Indigenous women included higher rates of substance use, depression, and suicide attempts [7, 9, 26, 27]. Revictimization was also commonly reported for Indigenous survivors of childhood sexual abuse [7, 9]. Rates of lifetime PTSD are disproportionately high in Indigenous populations, likely due to higher overall exposure to trauma, including sexual trauma [4]. Indigenous women survivors of sexual assault have also demonstrated protective outcomes after assault, including higher rates of help-seeking and increased use of traditional healing methods [27]. Despite these findings, scholars have repeatedly identified a need for more research on the risk and protective factors of sexual violence in the lives of Indigenous women specifically [67, 69].

There are also a limited number of qualitative studies examining the lived experiences of Indigenous women who have been sexually assaulted. One study of Cheyenne women identified some common immediate reactions to sexual assault including feeling shame, embarrassment, anger, and degradation, with women living at home likely to hide the assault from their family [6]. Further, most survivors (96%) in this study did not include the assault in their life story, preferring to remove, or forget, the incident from their life [6]. Another qualitative study of Canadian Indigenous assault survivors identified which emotional, physical, mental, and spiritual sources of healing were most helpful post-assault [45]. Additionally, a two-generation qualitative study of Indigenous families enabled parents and their adult children to process their own experiences of sexual abuse and its impact on their own life histories [53]. However, experiences of sexual assault in Indigenous communities are often silenced [9] and understudied [23], and therefore, more research should be conducted which gives voice to the lived experiences of Indigenous survivors of sexual violence.

## The FHORT’s Resilience and Transcendence: Relational and Spiritual Protective Factors

While not studied as extensively as risk factors, resilience, and protective factors (those that buffer against negative outcomes) in recovery related to sexual assault have also been studied [25, 61, 62]. Among the general population, social support has been identified as a key protective factor in the prevention of sexual assault [62]. After an assault, social support, especially from friends, relatives, and the community are associated with better outcomes [25, 61, 65]. However, this social support relationship was strongest when the survivor did not use substances to cope [25] and reported lower rates of loneliness [65]. For childhood sexual abuse survivors, family support, family cohesion and lack of family conflict are associated with improved resilience and recovery after assault [46]. Moreover, high levels of spirituality after an assault were associated with increased well-being, particularly for racial and ethnic minorities [43].

## Contemporary Forms of Historical Oppression: Barriers to Justice for Violence Survivors

Perpetrators of sexual violence against Indigenous women include non-Indigenous, Indigenous, and even family members. In some communities, sexual violence against Indigenous people is disproportionately committed by interracial perpetrators [1, 2, 23, 58], and this depends on the region and context of each tribal nation. In such communities, Indigenous men and women are approximately three times as likely to be assaulted by an interracial perpetrator when compared to White men and women [58] and an estimated 57 percent of sexual assaults committed against Indigenous women are perpetrated by White offenders [2]. Using the FHORT, these high rates of interracial sexual assault in particular can be understood as part of the ongoing legacy of colonization.

The 1978 decision in Oliphant v. Suquamish ended tribal jurisdiction over non-Indian perpetrators of sexual violence on Indian country, requiring Indigenous survivors of violence to rely on the federal government for justice [23, 57]. As approximately 67 percent of sexual violence crimes deferred to federal prosecutors are declined, this results in a severe miscarriage of justice for Indigenous women [33]. In 2013, the Violence Against Women Act was updated to extend tribal jurisdiction to sexual violence perpetrated by intimate partners but did not address stranger or acquaintance sexual violence [23, 33]. A 2019 VAWA Reauthorization Act seeks to close this loophole but has not passed at the Senate level [8]. This loophole leaves Indigenous communities particularly vulnerable to what Smith called “legal rape” (p. 39) [63].

Not all sexual violence is perpetrated by non-Indigenous individuals. Due to the internalization of patriarchal norms that devalue women, many tribal members and even families target women who they are close to. Societies which once valued and revered women have adopted misogynistic norms that lead to a higher rate of violence [13]. One study of six tribes found that woman survivors were most commonly victimized by male relatives (55 percent) and romantic partners (46 percent) [72]. The ongoing legacy of colonization has also interrupted processes of justice for those victimized by Indigenous individuals. For instance, the Major Crimes Act (1885) and Public Law 280 (1953) eliminated tribal sovereignty in responding to sexual violence, and the Indian Civil Rights Act of 1968 severely limited the extent of sentencing allowed in tribal courts [23]. This sentencing cap was later extended through the 2010 Tribal Law and Order Act, but many are pushing for the full restoration of tribal jurisdiction and the necessary funding as an important step towards justice for survivors of sexual assault [23, 57].

The purpose of this multimethod study was to use the FHORT to qualitatively examine Indigenous women’s experiences of sexual violence and examine how sexual violence differentiates across key outcomes from a wellness perspective. Indigenous women are particularly susceptible to violence in the U.S. As such, this inquiry contributes to the understanding of Indigenous women’s experiences of sexual violence, as well as to understanding the extent that experiences of sexual violence may contribute to key aspects of wellness. Understanding the potential for sexual violence to be a driver of key aspects that affect health disparities is essential to addressing underlying mechanisms that perpetuate health inequities for Indigenous women. The overarching research question for the qualitative component is: What are the experiences of sexual violence within these tribal communities? This question informed the quantitative inquiry: How do key outcomes from a wellness perspective differ between sexual violence survivors and those who do not report sexual violence? Fig. 1 displays the FHORT and key outcomes examined in this inquiry. We examined gender differences under the hypothesis that sexual violence would be higher for women.

## Methods

### Research Design

Using an exploratory sequential multimethod design, we prioritized qualitative data from 436 participants. These results informed the quantitative data collection and analysis which included 127 survey responses. Survey instruments were designed to measure key themes identified in qualitative data. The research team developed surveys to directly assess how emergent qualitative themes predicted key outcomes related to resilience. With a combined total of 563 participants, the data sources mutually informed each other in an iterative data analysis process [21]. These data emerged as a part of a critical ethnography exploring the lived experiences for Indigenous peoples in the Southeast and culturally relevant risk and protective factors related to intimate partner violence (IPV) and alcohol and other drug (AOD) abuse [17]. Triangulating many forms of data, as done in this critical ethnography, enables greater rigor [13]. Further, using critical theory, power differentials between colonized and marginalized groups are explored in critical ethnography [19].

To conduct ethical and culturally relevant research with Indigenous peoples, recommendations from the “Toolkit for Ethically and Culturally Sensitive Research with Indigenous Communities” were followed [17]. Table 1 presents these strategies [17]. The first author has become educated and worked with the focal tribal community for over a decade, she is embedded in the community, works with extensive cultural insiders, uses culturally appropriate methodology with storytelling and tribal analysts and research assistants, builds upon cultural strengths and uses a tribal perspective. Additionally, investment in the community, advocating, and giving back is demonstrated through this inquiry informing the development of a culturally and community-based intervention that addresses sexual and family violence and substance abuse in communities, while promoting family resilience and wellness [52]. These strategies further fulfill the call for trauma-informed research [70] as they align with the five principles of trauma-informed practice: safety (honor confidentiality, be transparent), collaboration (commit long-term, collaborate), choice (enable self-determination, listen), trustworthiness (build a positive reputation, spend time in the community), and empowerment (reinforce cultural strengths, invest resources) [29]. For a full description on how these strategies were integrated in this study, see McKinley, Figley, et al., 2019 [50].

Ethnographic methodologies are characterized by incorporating multiple forms of data to inform central research questions, including interview data and survey data [19]. First, we integrated data across the two tribes from three sources: focus groups, family interviews, and individual interviews with (a) youth (ages 11 to 23); (b) adults (ages 24 to 55); (c) elders (ages 60 and older); and (d) behavioral health professionals. We assessed the same qualitative theme, triangulating across different age groups and interview modalities to add to the credibility and consistency of results across ages and interview types. Next, we recruited tribal members across communities to participate in a quantitative survey to assess and validate whether qualitative themes predicted key outcomes of interest related to sexual violence. We focus on data related to sexual violence across all sources.

### Setting

To protect community identity, the names and identifying information of the two Southeastern tribes are confidential [17]. “Inland Tribe” and “Coastal Tribe” are used as pseudonyms to protect community identity. The Inland Tribe is in proximity to the Gulf Coast and federally recognized. It includes tribal services including schools, healthcare facilities, family violence and social services, and justice and law enforcement. The Coastal Tribe is also on the Gulf Coast and is state recognized but has not received federal recognition. The Coastal Tribe has limited employment and education services.

### Data Collection

Both Institutional Review Board and tribal approval for the study were obtained. Participants were recruited through word of mouth, online (through flyers) and in person (through agency leaders and cultural insiders) [17]. For primary data collection, out of cultural sensitivity, a tribal interviewer was offered as an option [17] but participants felt more comfortable speaking to someone outside the tight-knit community. Therefore, the first author collected the data [17]. In all, 27 focus groups (217 participants), 64 family interviews (163 participants) and 254 individual interviews were conducted. Participating individuals and families received $20 and $60 gift cards respectively to local department stores. The semi-structured interview guides were developed with the help of cultural insiders and written at a fifth-grade level for comprehension. An example of a semi-structured interview question related to this inquiry was: “Some people have experienced being abused sexually. Describe any memories of these hard times. How do you think experiences of violence have affected you?” A life history approach for Indigenous peoples was followed for individual interviews [19] and participants each received copies of their interview transcript and a summary of results. Focus group and family interview guides followed the same topics of the life history interview but in a more general way. The focus of this work was to find unifying themes across interview types and participants. The themes were consistent across interview types, with group interviews tending to go less in depth due to the nature of interviews. Group interviews extended and confirmed content that was explicated in a more detailed way in individual interviews.

Tribal community members (including but not exclusive to the qualitative participants) were invited to participate in an online Qualtrics survey as part of the quantitative data component. All of the survey participants were entered into a drawing and over half received a $50 gift card for their participation. Approximately 80 percent (*n* = 127) of those starting the survey (*n* = 161) completed the survey. Participant demographics are included on Table 2.

### Qualitative Data Analysis

For qualitative data analysis, team-based analysis methods were conducted, including tribal and non-tribal research assistants [37] and following the guidelines recommended by the “Toolkit for Ethically and Culturally Sensitive Research with Indigenous Communities” [17]. Beyond the first author, six team members received in-depth training including educational readings, the use of the qualitative software, and data analysis training. Team members shared coding examples of transcripts with explanations of coding during training sessions to answer questions and explicate the theoretical framework. Each team member practiced on several example transcripts before coding independently. Transcripts were reviewed by the first author, and at least two members coded each transcript to ensure interrater reliability and credible arrival at results.

Team members completed analysis on a timeline in which multiple team members reviewed transcripts for increased trustworthiness of findings. Likewise, because at least two team members were coding simultaneously, they were able to utilize peer support for any questions. Each team member recorded any questions, codes added, and communication on a coding log that was shared among the team, which served as an audit trail. The analysis team met bi-weekly throughout data analysis to discuss interpretations, questions, and engage in dialogic discussion of results. Interviews were professionally transcribed and analyzed, with one NVivo data analysis software file for each tribe. Using a reconstructive thematic analysis approach, research team members listened to and read interviews at least twice for immersion in the data. Next, in consultation with team members, the first author created hierarchical structure of themes and subthemes from low-level coding. All of the research team reviewed themes for cultural appropriateness, and their feedback was incorporated into the hierarchical coding.

The final stage of the analysis was to uncover the implicit and explicit meaning of primary codes, leading to final coding structures. For instance, after line-by-line coding uncovering surface-level themes, sections were selected to examine for deeper, underlying, or more abstract meanings. Meanings were discussed with the tribal context and cultural protocols in mind, and final themes reached by consensus. Cohen’s kappa coefficients [48] was extremely high (i.e., 90 or higher) indicating interrater reliability. This article focuses on the unifying themes concerning how people experience sexual violence. Tribe, gender, and category (e.g., focus group, family interview, or individual interview) of participants are included in the results for reference. Codes related to this theme emerged from over 200 (*n* = 205) Inland and Coastal tribal participants.

Over half of participants engaged in more than one interviews. Participants received a summary of results, a transcript of their individual interview(s) and an invitation to edit or elaborate on the findings and transcripts as a mechanism of member checking. Many participants took the opportunity to elaborate and all participants who could be reached supported the findings. On more than 10 occasions, the PI disseminated results to the community through dialogues, trainings, agencies, tribal councils, and committees. Throughout the data collection and analysis period, research team members participated in weekly peer debriefing.

### Quantitative Data Analysis

The quantitative inquiry focused on identifying differences across ecological aspects of wellness among individuals who experienced sexual violence versus those that had not experienced sexual violence. Listwise deletion was used for missing data [42]. and no problems with multicollinearity among independent variables were identified. In total, 11 participants were missing data for key outcomes, leaving 116 in the final analyses. We performed all analyses using SPSS Version 27 (see Table 3 for a list of measures and their descriptions). Next, significant differences between sexual violence survivors and those not reporting such violence were examined using *t* Tests across the following key dimensions of wellness: (a) structural: historical oppression, historical loss, oppression, and discrimination; (b) relational: adverse childhood experiences (ACE) and stressful life events, family resilience, and social support; (c) spiritual: spiritual-well-being and life satisfaction; and (d) psychological/behavioral: alcohol use, posttraumatic stress disorder, and resilience. We also calculated effect sizes for each key outcome (See Table 3).

## Results: Qualitative

Qualitative themes describing experiences of sexual violence focused on younger girls and women being targeted, largely by family members, older males, and males in general. The perceived accountability and justice for these crimes was reported as insufficient or nonexistent. The themes we now investigate in detail are: (a) “It’s mainly within the family”—Familial Perpetrators of Sexual Violence; (b) “How many people can I sleep with?”— Non-Familial Perpetrators of Sexual Violence; and (c) “You can get away with anything here … No Justice at All”— A Lack of Accountability for Perpetrators. The focus now turns to these themes.

### “It’s Mainly within The Family”— Familial Perpetrators of Sexual Violence

Following the FHORT and the imposition of patriarchal gender roles leading to violence against women and girls, vulnerability of women and girls to sexual violence at the hands of a family member was discussed by many participants. A woman IPV provider from the Inland tribe noted in a focus group the gender of most of the survivors they worked with: “For us it’s mainly girls, I think we only have less than five that are boys.” The Inland tribe is largely self-containing with less interactions with outlying non-Indigenous communities. As such, an Inland IPV woman professional from a focus group explained: “I’m going to say it’s mainly within the family…Usually it’s the parents, either their boyfriend … or husband doing the sexual abuse with kids.” Sexual harm to a family member represents a reversal of the female-centered Indigenous traditions that held women and girls in high status, to a dehumanized role where they are targets of sexual violence. This violence tended to be intergenerational. A woman from the Inland tribe’s mother’s previous experiences of familial sexual violence led her to be protective:

So, so overall [mother] she kept me protected. … I finally figured out, when I was growing up that, the reason why she raised me the way she raised me was… she was raped. … Then the other time before when she was growing up, she was just a young girl. It was an aunt’s husband that tried to get in bed with her, and she kicked him off and got up. But the aunt told her “Don’t tell, don’t tell Grandma, don’t tell Grandma. She’s going to be mad.” I mean you don’t do that…And that’s [how] come [*sic*] she raised me the way she raised me. Everything was sheltered, if I’m going anywhere, all my brothers, I’m the second but I got a younger brother that’s two years older but everywhere we went, I mean, my world was sheltered.

This woman’s mother was determined to protect her from experiencing the same form of abuse she had; however, this participant’s partner ended up perpetrating sexual abuse on other family members. Sexual abuse experiences perpetrated by male family members was described by several speakers, including one woman youth participant from the Inland tribe who was abused by multiple family members:

It’s not easy to spot out who, like, the sexual predators are. …When I was about 7 or 8 … my cousin and my uncle [committed sexual abuse], but it’s happened at two different times. … I would go over to my grandpa’s house. My uncle lived with my grandpa. Uh, before my grandpa passed, and, um, he would kind of bring me in his room, and touch me over my clothes, and then it would just make me uncomfortable. … I really didn’t know what he was doing … and so … and then after he got done, he just told me to go sit in the living room … I just didn’t tell anyone. Like, he didn’t threaten me. I was just scared that he was going to get in trouble for what he did

In part because they were family members, this speaker felt she needed to protect the abuser from harm and didn’t tell anyone about the abuse until several years later.

### “How Many People can i Sleep with?”— Non-Familial Perpetrators of Sexual Violence

Perceiving women in dehumanizing ways – as sexual objects of conquest to exploit – was spoken about by some participants. As evidence of the internalization of patriarchal gender role ideology, a woman Coastal participant expressed this shift from respectful to disrespectful views of women. She stated:

Before they [males] were more respectful. [Now], it’s more of, “How many girls can I sleep with?” instead of finding that one girl and then staying with her. It’s “How many people can I sleep with?” And they disrespect the women. Girls, they’re actually girls, and the girls feel like they have to do it because they want the guys to like them. It was kind of bad whenever I was growing up, but it seems like it’s even worse now.

A male youth from the Inland tribe explained how he perceived women as objects of conquest. He stated, “I don’t call them my girlfriend.” When characterizing how males and women in his school go together, he stated, “Yeah, they hook up.” He went on to add, “I’ve had a lot of girlfriends –Hooking up.” When asked, “Do you worry about that like what if they had a kid or something like that?” He replied, “I don’t care. Because if I’m done with a person, I guess I ain’t [*sic*] talking to them.” When the interview replied, “They serve their purpose type of thing? That’s interesting. How do you think they feel after that?” He repeated, “I don’t care.” Despite this youth wanting to “impress” with his lack of empathy, this male, with a history of drug, school, and family problems, described a pattern of objectifying and using women for sexual pleasure, and then casting them aside, regardless of the consequences or implications.

A pattern of older males seeking out younger women for intimacy and sex was frequently described. A Coastal elder woman described conflict across past and current relationships. She described her ex-husband who experienced legal consequences for relationships with underage girls, “First he went to jail for child support, then it was for beating up his girlfriend.” She was now married to a person where infidelity was a problem. She had been married for a decade and she explained, “He’s not a good person—lying, cheating.” When asked, “When did things start to turn south?” She replied: “About maybe a year after we was married. He started cheating.” When asked, “How did you find out?” She replied, “He went to jail for it because it was an under-aged person. … It’s carnal knowledge.” Young girls are also often portrayed as being targets for older males in the community. One woman youth from the Inland tribe described being propositioned in a Walmart parking lot:

Older men, I would say, maybe like in their 20 s, or something, are still coming at me.

I was like, I was like, like recently, like, I was with my best friend. We were in Walmart, and this black guy up came to us, and he asked if we were looking for like, older, older men to date…He was referring to himself, and, um, I was like, “Uh, no,” and so…

He, he was walking away, but he turned back around, and he looked at us, and he came back, he goes, “Y’all sure you’re not looking for anyone,” so we were like, “No,” and so, he put his arm around my friend.

Another woman youth from the Inland tribe described the frequency of girls being assaulted: “There’s been a lot of rape in… [the] neighborhood.” An additional woman youth (aged 20) from the Inland tribe described seeing men taking advantage of young girls and being concerned about the safety of her own daughter “There’s too many guys that just take advantage of little girls a lot. I see that a lot… that’s what scares me sometimes, like because I have a daughter of my own.” Another woman IPV professional recalled being at a party at a young age and almost being assaulted while sleeping:

It was like a drinking party. And I… was wanting to sleep. And it was at a certain house in another community. And this lady noticed…and she said, “Are you sleepy?” I said, “Yes.” And she’d said, “Well, you can sleep in this room, but make sure this door is locked.” I slept. Next thing I know, this man was on top of me. And like I said, he was drunk, and so I kinda pushed myself out… and ran to the car and locked myself in…I was about maybe 9, 10, 12.

As indicated by this participant, girls could be targeted at as young of an age as nine or ten.

### “You can Get Away with Anything Here … No Justice at All”— A Lack of Accountability for Perpetrators

Many participants spoke about violence and sexual abuse targeting young girls that was exacerbated by the lack of accountability for the perpetrators in the criminal justice and law enforcement systems. A youth woman from the Inland tribe experienced abuse at the hands of family members: “My aunt’s, um, her husband had been taking pictures of my sister when she’s sleeping, and she didn’t notice that … And finally, she came out when she realized what he was doing.” This speaker went on to describe the lack of accountability in the tribe and how she desired that law enforcement do more to address violence in the community:

When you asked me like, what should I [be] change[d] in in the tribe. It’s law enforcements mainly… You can get away with anything here…You kill a person; you get to walk the streets. You- you see somebody die and you just walk away just thinking there’s nobody gonna help them…No justice at all…Nothing happened…We- we reported it [the sexual assault] and they never got back to us. Our law enforcement is very low and no [justice] … Like, I could honestly just go out here and kill somebody and I would just get released.

This woman in high-school described a lack of justice in the court system for her sexual violence:

I think they [perpetrators] should, uh, be in jail for what they have done. Well, not jail, maybe prison…As long it’s something not, like, here at the [name of local jail]. It’s like a walk in the park. … Like when my mom came back…they called the police… He [familial perpetrator] came back to the house, and, um, back then they didn’t arrest him … He ended up in jail, but, you know, he didn’t stay in there for very long …What makes me sick is that they didn’t keep [him] in jail that long. I mean … What if he got another girl? I was like, another little girl maybe about that age, and I was like, maybe the [justice organizations] will learn to listen and keep them in there longer … but the thing was my friend was like, his niece, had like kind of beat me up, just because I put her uncle in jail.

This speaker not only describes her frustration at the short sentence the perpetrator experienced, but also describes being punished and ostracized by community members for coming forward and reporting the assault. In contrast, a woman professional in IPV services reported that her family member was held accountable by other family members for attempted sexual assault:

I had almost got raped twice…Luckily, I didn’t because they were drunk. … I was small enough to get away from their hands. And one of them was … Actually, she [family member] was married to one of them…And my uncle had stood up for me and said, um, “Either you divorce him or he’s gonna rot in jail”…So she took, you know, the divorce.

In this case, the speaker was able to get support from her family and the abuser was kicked out of the family after she reported what happened to her. However, this experience still highlights the vulnerability to sexual abuse young girls experience from male family members. Another youth woman from the Inland tribe described being assaulted at a young age in public:

Now I just don’t feel safe …It was at my little sister’s softball game…and there was this umpire. And I’ve always talked to him, but I never knew he was like a pervert or anything. And well, um, he put his arm around me, and I just didn’t think it was anything. And that’s when he started… he didn’t go into my shirt, but he was like over my shirt. And he was just touching my left breast, and I didn’t do anything. And then that’s when [I] just told him I was going to my truck. And then, like a couple weeks later, like two [weeks] … I told my mom, and then we just started filing charges, but like it’s been a year already that they haven’t done anything about it. …It makes me mad because … it’s been so long, and they’ve waited on everything…They let him [abuser] be around there still…And work.

Not only did this speaker not get justice from the courts, but she was also forced to continue to see her assailant at public events, where the perpetrator could access other victims.

This vulnerability of young girls also extended to teen relationships. A woman practitioner described the vulnerability teen girls experience in dating relationships, particularly because law enforcement often appears disinterested in pursuing these cases:

I can’t believe how high the dating, the violence…They’re teenagers…We just have them come in and just have [*sic*] to talk to them…I look for safety issues … You’re going to come across officers that has [*sic*] an attitude … talking like I don’t care, I don’t want to listen to it. I’m like, why is he [officer] there?

Despite the practitioner noting the increase they saw in teen dating violence, the court justice system seemed to be not set up to handle cases of adolescent IPV. Other barriers in the justice system included victims being expected to testify on the stand, as one practitioner noted:

I think if victims didn’t have to be on the stand a lot of cases would [go forward]… I know a lot of people don’t agree with that, but I would because if you are sitting there telling your story and your perpetrator is right there … this person still has control over you…You’re looking right at them … I wish they could be able to do that like you said either give a statement or have that person either tape recorded or video.

Girls were often described as being at increased risk for sexual violence, both in the community and at the hands of male family members. The justice system was often cited as a barrier for seeking and achieving justice when a physical or sexual assault took place. Thus, results indicated an internalization of patriarchal norms of sexual conquest toward women, exacerbated by impunity for perpetrators.

## Results: Quantitative

We assessed whether participants who experienced sexual violence perceived historical oppression, loss, and oppression to be prominent, as well as how they experienced support and stressful life events and resilience. We examined how they differed in relationship to key outcomes of health disparities, namely alcohol use and PTSD. To identify differences across ecological aspects of wellness among individuals who experienced sexual violence versus those that had not experienced sexual violence, independent *t* Tests indicated significant differences on several dimensions of wellness including (a) structural: higher historical oppression, historical loss, oppression, and discrimination; (b) relational: higher ACEs and stressful life events and lower family resilience and social support; (c) spiritual: lower spiritual-well-being and life satisfaction; and (d) psychological/behavioral: higher levels of alcohol use, posttraumatic stress disorder, and lower levels of psychological resilience. Table 4 displays *t* Test results examining these hypotheses.

All of these hypothesized relationships were confirmed, namely that survivors of sexual violence perceived higher historical oppression, historical loss, oppression, discrimination, ACEs, stressful life events, alcohol use, and PTSD symptomatology. Moreover, reported survivors of sexual violence reported lower levels of family resilience in their upbringing, life satisfaction, psychological resilience, spiritual well-being, and social support. The effect sizes for all outcomes ranged from medium to high (0.52–1.14). Indeed, historical oppression, historical loss, life satisfaction, psychological resilience, spiritual well-being, and social support all had at least a medium effect size, whereas oppression, discrimination, ACEs, stressful life events, family resilience, alcohol use, and PTSD all had large effect sizes.

## Discussion

This research provides perspective on the devastating experiences of sexual violence felt by Indigenous women and girls, lack of accountability for perpetrators, as well as the profound impact across major outcomes of interest of wellness and health disparities. Past research indicated forms of historical oppression and losses leading to the replacement of humanizing, matrilineal roles with dehumanizing, patriarchal gender ideology that placed women at risk of violence. This same research also coupled this historical loss with a lack of accountability for perpetrators, as people tended to keep silent about violence [13].

Qualitative participants extended these sentiments, reporting surviving sexual violence at the hands of both familial and non-familial perpetrators. Internalized patriarchal colonialism was evident with the frequencies with which participants reported family members targeting the women and girls in their families for sexual violence. Unlike other tribes with greater interaction with non-Indigenous communities and because of the context of the focal tribes [1, 2, 23, 58], where there was limited exposure to outside communities, family perpetration may be higher. These findings are consistent with the literature, which found that across six tribes, 55 percent of woman survivors of sexual assault were victimized by a male relative [72]. Having familial perpetrators of sexual violence complicated feelings, with some survivors feeling the need to protect or not report this violence due the perpetrator being a family member. This reaction is common as one study found that only over 60 percent of Indigenous sexual abuse and violence survivors ever tried to get support [45]. Specifically, many ethnic minority young girls do not report sexual assault due to fear of racial bias resulting in disbelief of victims and harsh punishments for perpetrators [9].

Sexual conquest was not only a tool used in colonization, but it was also perpetuated in the present [12, 13, 16, 34]. Dehumanizing beliefs placed women and girls at risks, being perceived as objects of sexual desire. Participants potentially internalized patriarchal gender norms introduced in colonial historical oppression [12, 13]. Sexual violence was seen as a tool of colonial conquest [12] and seeing “how many girls can I sleep with?” was a perception that was normalized and internalized within some community members. Older men targeting and “taking advantage” of younger women and girls was also remarked upon, with participants being assaulted as early as nine or 10. This young age of assault is particularly troublesome due to the high rates of revictimization of childhood sexual abuse survivors [9, 61]. Moreover, justice in the court system was unsatisfactory as stated, “You can get away with anything here.” Consistent with extant research [13], instead of accountability and justice, participants reported perpetrators getting away with sexual violence with impunity, a topic that has been supported by cross-national research [1, 23, 63]. Parallel to this research, perceived accountability or justice for these crimes was reported as insufficient or nonexistent [1, 13].

Not only were experiences of sexual violence deeply consequential in qualitative research, but quantitative results also indicated sexual violence survivors had differential outcomes across structural, relational, and personal levels. Supporting extant research [18, 46, 61] results reveal sexual violence had extensive impacts across multiple areas of life. Despite being exploratory, all outcomes had medium to large effect sizes. Quantitative findings revealed that experiencing sexual violence profoundly differentiated across all dimensions of wellness. First, experiencing sexual violence was associated with significant differences related to historical oppression, on the structural dimension of wellness. Participants who reported sexual violence also reported higher perceived historical oppression, historical loss, oppression, and discrimination. This was an interesting finding in that sexual violence survivors perceived (and by results also experienced) greater contemporary and historical oppression and discrimination. Because sexual violence was a pivotal aspect of colonization, it could be that woman Indigenous survivors sit at the intersection of cumulative forms oppression, where they have been targeted as women and young girls and as Indigenous peoples [1, 4, 7, 9, 13, 16].

Second, at the relational level, sexual violence survivors reported higher levels of ACEs and stressful life events and lower family resilience and social support. Historical oppression dramatically undermined Indigenous families. Boarding schools systematically removed Indigenous children from their families and communities –precluding their ability to love and socialize them and rather assimilating and exposing children to environments replete with sexual abuse and exploitation [68]. Consistent with research indicating the intergenerational experiences of violence, surviving sexual abuse in childhood increased the likelihood of experiencing such violence as an adult [61]. Given many experienced abuse by a family member, it is no surprise that higher levels of ACE and stressful life events would be higher. Indeed, Indigenous women are exposed to higher levels of trauma than other populations in general [4] and this trauma contributes to a pile-up or an accumulation of risk factors from the resilience perspective. Exposure to trauma and PTSD tend to be elevated among Indigenous peoples, up to two and three times that of non-Indigenous peoples [3, 5, 41]. Moreover, the protective factors of family resilience as well as social and community support were also less for sexual violence survivors; this would exacerbate recovery from sexual violence and contribute to poorer outcomes, including substance abuse and mental health [25, 61, 65]. Indeed, and as expected, at the psychological level, sexual violence survivors, on average, experienced higher consumption of alcohol and PTSD – both disparities driving the mortality and morbidity of Indigenous peoples [18, 52, 61].

Finally, and of utmost concern, sexual violence survivors reported a lower quality of life, resilience, and transcendence across a plethora of measures. Regarding resilience and transcendence, sexual violence survivors reported lower levels of psychological resilience, life satisfaction, and spiritual well-being. Experiencing sexual violence seemed to cut to the core of women’s well-being and potentially constrained their sense of self and wellness. Given that psychological resilience contributes to people experiencing mastery and control in their world, sexual violence may tend to strip women of these protective perceptions, at least for a time. Further, the lack of justice for survivors of sexual violence may act as a further roadblock to resilience, as injustice may contribute to feeling helpless and out of control. Life satisfaction was also lower for violence survivors, along with spiritual well-being, which could connect to violence survivors feeling loneliness and disconnection [65].

## Limitations and Future Research

Results from convenience samples cannot be extended beyond their context. Variables and surveys were self-report measures rather than direct observations, and the survey measure for sexual violence was within the context of intimate partnerships. To follow IRB protocol, identifiable information was not kept for the survey portion of the study. As such, despite some participants from qualitative component also likely participating in quantitative component, we were not able to track who participated in both portions. Assessments of lifetime sexual violence and assault are likely higher. This research was cross-sectional. Although it displays a snapshot of how people were coping and experiencing life at the time of data collection, it did not capture how Indigenous women were faring over time, where longitudinal analysis would be helpful. With the heterogeneity of Indigenous Nations, results could benefit from replication and examination across additional contexts for a broader understanding. This research was exploratory only. Still, robust medium to large effect sizes indicate that results warrant further investigation to validate, replicate, and extend results. Future research should investigate the preliminary connections identified between historical oppression, loss, and discrimination with sexual violence. This complex relationship could benefit from some close inquiry.

## Conclusion

Indicating continued forms of historical oppression, this article provides personal experiences of the devastating and helplessness felt by survivors of sexual violence who experienced dramatic consequences, while they often witnessed their perpetrators walk away with impunity. Experiencing sexual violence also meant that survivors experienced higher levels of historical oppression, loss, discrimination, ACEs and stressful life events, with lower family resilience, life satisfaction, resilience, spiritual well-being and social support. They also reported higher levels of alcohol use and PTSD. Thus, sexual violence seems to be a pivotal point around which a host of risk factors accumulate.

To our knowledge, this is the first inquiry to examine connections between structural (historical oppression, loss and discrimination) relational (ACE, stressful events, social support, and family resilience) psychological and behavioral (psychological resilience, as well as alcohol use and PTSD) and spiritual/transcendence factors related (spiritual well-being, psychological resilience, and life satisfaction) to sexual violence in a rigorous multimethodology with Indigenous women. Connecting these structural, relational, contextual, and psycho-spiritual aspects as they relate to sexual violence and finding such robust findings in this preliminary research is striking.

The extent to which sexual violence cut across these dimensions of wellness is in some ways haunting, indicating the strong need to prevent, treat, and address the ecological risk factors in a holistic way. First, historical oppression, loss, and discrimination need to be redressed through decolonizing the patriarchal roles that were imposed and reversed what were protective social norms for many women in Indigenous communities –those that were matrilineal and woman-centered (matrilocal) [12, 13, 16]. Consciousness raising groups for Indigenous communities to self-determine pathways towards liberation and transcendence are implicated, particularly those with women who have survived sexual violence at the decision-making table. Re-envisioning gender roles in what once were complementary and empowering versus the adversarial roles imposed by colonization may be a topic of these groups. Moreover, enhancing social support and spirituality should be prioritized as both have been identified as key elements of positive outcomes after an assault for Indigenous women [9]. Indigenous culture has also been identified as a key source of healing [45]. One study of Canadian Indigenous people proposed using the Medicine Wheel as a way to holistically heal from sexual assault and violence by addressing spiritual (cleansing ceremonies, meeting with elders) emotional (connecting with peers, elders, family) physical (fasting, sobriety) and mental (learning language, counseling, self-help) domains [45]. Finally, this study further emphasizes the need to decolonize processes of justice to reinstate safety and security for Indigenous women and survivors.

There is a clear need for further study on the pathways to recovery or resilience [23] that incorporate culture, healing, family, and spirituality. Some research prioritized the voices of Indigenous women and professionals to identify solutions to sexual violence and other forms of IPV [16]; Survivors and professionals identified the importance of bolstering family and community resilience and supports [16]. McKinley, Figley, et al., (2019) [50] described the process of using community engaged and community-based participatory research to develop evidence-based or evidence-informed programs with the FHORT to redress historical oppression, bolster resilience, and reduce sexual violence and other types of violence and substance abuse in families [52]. Others can follow these models to work collaboratively with tribal communities who can clarify pathways towards violence-free transcendence and wellness.

## Figures and Tables

**Table 1:** Toolkit for Ethically and Culturally Sensitive Research with Indigenous Communities

Strategy for researcher(s)	Description
Become educated	Read about the specific and broad Indigenous history. Learn from Indigenous communities, colleagues, and insiders
Work with a cultural insider	This insider will lead the way to working within culturally appropriate protocols and nuances of the Indigenous community
Get invited	Collaborate with key insiders and become invited because of demonstrated skills and competence
Exhibit cultural humility	Approach work with Indigenous communities with a positive intent, authenticity, and respect for the people
Be transparent	Be completely open and honest about research intentions and resources available to do this work
Spend time in the community	Take the time to get to know Indigenous community members before beginning the study
Collaborate	Become embedded in the community and develop a network of people who conduct culturally sound research
Listen	Attend to Indigenous community members, whom are experts on their own community
Build a positive reputation	Build a reputation for doing worthwhile research
Commit long term	Work with Indigenous communities long term to foster lasting change and collaboration
Use a memorandum of understanding	Outline important guidelines such as who owns the data, how research findings are published, how researchers will follow-up with the community, etc
Use a cultural reader	A cultural reader reviews reports and prevents inadvertent harmful publishing
Go the distance	Travel to Indigenous communities, which might be a long distance away
Demonstrate patience	Understand that relationship, trust-building, and the research process take time
Enable self-determination	Incorporate the tribe’s input and participation throughout the research design and implementation
Use a tribal perspective	Avoid imposing a Western perspective
Use appropriate methodology	Use culturally congruent community-based, qualitative, or quantitative methods
Reinforce culture strengths	Build on the many strengths within Indigenous communities by using a community-based participatory method, and incorporating traditions in research such as storytelling, family, attention to land and the spirit, and other strengths already present
Honor confidentiality	Consider community, family, and individual confidentiality and how to ensure it, especially in tight-knit communities
Advocate	Communicate the needs and rights of Indigenous peoples to decision-making bodies
Reciprocate and give back	Develop programs, report results, provide compensation, and enable the Indigenous community to determine follow-up
Allow for fluidity and flexibility	Balance rigor with culturally congruent research practices. Adapt the research process to honor the community’s rhythm and traditions. Publishing institutions can support this flexibility as good research practice
Develop an infrastructure	Build a network with Indigenous and non-Indigenous researchers and community members to centralize and facilitate streamlined research that is useful for both Indigenous communities and academia
Invest resources	Funding sources can foster culturally congruent research by allowing for traditional customs, such as feeding participants or offering gifts to elders, through grants that can allocate funds to Indigenous communities, colleges, and infrastructure

Table reprinted with permission from [17]

**Table 2:** Qualitative and quantitative participant demographics

Participant demographics	Qualitative (*n* = 436)	Quantitative (*n* = 127)
Inland tribe	228 (52%)	80 (63%)
Coastal tribe	208 (48%)	47 (37%)
Men	149 (34%)	23 (18%)
Women	287 (66%)	104 (82%)
Age (range = 21–80 years)	*M* = 40	*M* = 46
Married (yes)	126 (29%)	51 (40%)
Children (range = 0–14)	*M* = 2.6	*M* = 3.8
Education	*n* = 307	*n* = 111
High School equivalent or less	147 (48%)	30 (27%)
Some college/Associates	116 (38%)	55 (50%)
Bachelor’s degree or higher	44 (14%)	26 (23%)
*Household*		
Single		15 (12%)
Couple		20 (16%)
Single-parent		25 (20%)
Two-parent		49 (39%)
Blended/Extended		18 (14%)
Full-time employment		85 (66%)
Fairly difficult to pay bills		69 (54%)
*Annual household income*		
< $25,000		39 (31%)
$25,001–$50,000		39 (31%)
> $50,001–$75,000		49 (39%)
*Community type*		
Reservation/tribal communities		105 (83%)
Nearby/off-reservation		22 (17%)

*M* mean; *SD* indicates standard deviation. Extended families include grandparents, aunts, uncles, cousins, etc. Blended families include stepparents and stepchildren. Table adapted with permission from McKinley and Miller Scarnato [51].

**Table 3:** Table of key outcome measures

	Variable and measure	Items	Response set	Example item (s)	Scoring	Reliability
NA	Sexual Violence -PVS	1	0 = no 1 = yes	Not including horseplay or joking around, My partner made me do sexual things when I didn’t want to	One item	(α = .87)
Structural Dimension (wellness)	Historical Oppression -HOS	10	0 = not at all 5 = a lot	As a result of historical events how much do you think members of your tribe have: taken out frustrations on each other and hurt each other through violence; Kept each other down	Added: Total Scores 0–50 with higher scores indicating higher levels of historical oppression	(α = .98)
	Historical Loss—HLS (adapted)	16	0 = never 6 = several times a day	Please indicate how often you think of these. The taking of our land; Loss of respect for elders by our children and grandchildren	Added: Total scores 0–96 with higher scores indicating higher perceived historical loss	(α = .95)
	Oppression—OQ	16	0 = not at all 3 = a great deal	My group is often looked down upon; We are treated as if we are inferior	Added: Total scores 0–48, higher scores greater perceived oppression	(α = .97)
	Discrimination—EDS	5	0 = never 5 = almost everyday	How often are: You are treated with less courtesy than other people; threatened or harassed	Added: Total scores 0–20 with higher scores indicating higher levels of perceived discrimination	(α = .84)
Relational/Social Dimension	Family Resilience FRI—Growing up	20	0 = no 1 = yes	Growing up (during the first 18 years of my life). Physical violence was not used or tolerated in my family; My family worked together to help each other around the house and to complete goals	Added: Total scores 0–20	(α = .92)
	Social/Community Support: SSI	17	1 = strongly disagree 5 = strongly agree	People in this community are willing to help & I have friends who let me know they value who I am	Added: Total Scores 17–85 with greater scores indicating higher social and community support	(α = .75)
	Stressful life events—LEC	16	0 = no 1 = yes	Indicate whether this happened to you: Natural disaster; Transportation accident; Physical assault	Added: Total scores 0–16 with higher scores indicating higher stressful life events	(α = .84)
	Adverse Childhood Experiences (ACE)	10	0 = no 1 = yes	Did a parent or other adult in the household often or very often Swear at you, insult you, put you down, or humiliate you?	Added: Total scores 0–10	(α = .79)
Spiritual	Spiritual Well-being—SHALOM	20	1 = very low 5 = very high	How important is developing a connection with nature; prayer life; meaning in life	Added: Total Scores 20–100 with higher scores higher perceived spiritual well-being	(α = .95)
	Life Satisfaction—SWLS	5	1 = strongly disagree 5 = strongly agree	In most ways, my life is close to my ideal; I am satisfied with my life	Added: Total scores 5–25 with higher scores indicating higher levels of satisfaction	(α = .90)
Psychological Dimension	Alcohol use: CAGE	4	0 = no 1 = yes	The extent a person thought they should Cut down, were Annoyed, Guilty, or had an Eye opener related to drinking/drug use	Added: Scores of 2 or above clinically significant	(α = .88)
	PTSD—PC-PTSD	4	0 = no 1 = yes	Have had nightmares about it or thought about it when you did not want to? Tried hard not to think about it or went out of your way to avoid situations that reminded you of it?	Added: Scoring “yes” to any three times indicates a positive screen for PTSD	(α = .87)
	Psychological Resilience—CD-RISC	10	0=not at all true 4=True nearly all of the time	I am able to adapt when changes occur; Having to cope with stress can make me stronger	Added: Total scores −40 with higher scores indicating higher levels of resilience	(α = .94)

Ace = Adverse childhood events [30]; CD-RISC = Connor-Davidson Resilience questionnaire [22]; CAGE = (cut down, annoyed, guilty, and eye-opener) [28]; EDS = Everyday discrimination scale [20]; FRS = Family Resilience Inventory [15]; HLS = Historical loss scale [71]; HOS = Historical oppression scale [49]; LEC = Life events checklist [35]; PC-PTSD = Primary care PTSD screen [56]; PVS = Partner victimization scale [38]; OQ = Oppression questionnaire [66]; SHALOM = Spiritual health and life orientation measure [31]; SSI = Social Support Index [47]; SWLS = Satisfaction with life scale [24]

**Table 4:** t-Test Results between those who reported sexual violence versus those who did not

Variable	Sexual violence *M* (*SD*)	No sexual violence *M (SD)*	*t*	*P*	95% CI for mean difference	*df*	Effect size
Historical oppression	38.47 (11.13)	30.59 (13.28)	− 2.31	.023	− 14.64, − 1.12	115	.61
Historical loss	48.88 (17.04)	40.30 (15.29)	− 2.10	.038	− 16.66, − .50	115	.55
Oppression	50.64 (10.85)	36.82 (14.71)	− 3.70	.000	− 21.22, − 64	115	.97
Discrimination	17.71 (7.22)	11.82 (4.84)	− 3.24	.004	− 9.70, − 2.07	18.5	− .98
Adverse childhood experiences	4.94 (3.82)	2.26 (2.02)	− 2.83	.011	− 4.67, − .69	17.6	1.14
Stressful life events	15.24 (9.90)	8.99 (6.73)	− 2.51	.022	− 11	18.6	.86
Family resilience (Growing up)	12.88 (7.79)	16.52 (4.11)	2.89	.005	− 10.01, − 2.48	17.6	− .76
Life satisfaction	20.71 (8.08)	24.51 (6.28)	2.20	.030	.38, 7.21	114	− .58
Psychological resilience	3.61 (1.00)	3.98 (.65)	1.98	.050	− .00, .74	114	− .52
Spiritual well-being	3.62 (.77)	2.98 (.67)	1.96	.052	− .00, .71	115	− .52
Social support	47.77 (7.64)	43.11 (8.94)	2.26	.025	.58, 8.74	115	− .59
Alcohol use	1.53 (1.81)	.55 (1.13)	− 2.16	.044	− 1.93, − 0.03	18	.79
PTSD	2.88 (1.58)	1.49 (1.65)	− 3.24	.002	− 2.2	115	.85

Sexual Violence = those who reported they experienced sexual violence by a current or former partner. No Sexual Violence = those who reported they had not experienced sexual violence by a current or former partner. Also tested but not significant were the variables measuring family resilience in the current family, depressive symptoms, anxiety symptoms, drug use, gender role attitudes, domestic violence blaming, and enculturation. *CI* = Confidence Interval; *M* = Mean; *SD* = Standard Deviation; *df* = degrees of freedom; *p* = probability or significance level
